# Following autophagy step by step

**DOI:** 10.1186/1741-7007-9-39

**Published:** 2011-06-02

**Authors:** Tom Egil Hansen, Terje Johansen

**Affiliations:** 1 Molecular Cancer Research Group, Institute of Medical Biology, University of Tromsø, 9037 Tromsø, Norway

## Abstract

Autophagy is an evolutionarily conserved lysosomal degradation route for soluble components of the cytosol and organelles. There is great interest in identifying compounds that modulate autophagy because they may have applications in the treatment of major diseases including cancer and neurodegenerative disease. Hundeshagen and colleagues describe this month in *BMC Biology *a screening assay based on flow cytometry that makes it possible to track distinct steps in the autophagic process and thereby identify novel modulators of autophagy.

See research article: http://www.biomedcentral.com/1741-7007/9/38

## 

Eukaryotic cells degrade proteins through two systems, the ubiquitin-proteasome system, and autophagy, whereby cytoplasmic components become enclosed in a double membrane to form a compartment known as the autophagosome and are delivered to the lysosome for degradation. Constitutive, basal autophagy occurs under nutrient rich conditions and serves as a quality control mechanism for both proteins and organelles, to protect the cell from the consequences of aggregated proteins and damaged organelles that could cause the development of disease: failure of this system is implicated in the development of, for example, neurodegenerative disease and cancer. Autophagy is also induced in response to starvation in order to generate nutrients and energy through the degradation of macromolecules and organelles [1,2].

## Autophagy and disease

Research during the last decade has made it increasingly clear that autophagy plays important roles in most of the major human diseases as well as in infection and immunity [1,2], with increasing evidence for selective autophagy of protein aggregates, organelles and pathogens [3]. Autophagy may, depending on the circumstances, either inhibit or promote tumor growth. Thus it may contribute to genomic stability by clearing cells of damaged organelles and protein aggregates that produce reactive oxygen species resulting in DNA damage; or on the other hand, it may help tumor cells survive stress conditions like hypoxia and nutrient deprivation [4]. Neurodegenerative disorders such as Alzheimer's, Huntington's and Parkinson's disease are characterized by the deposition of intra- and extracellular proteins aggregates that are not degraded by proteasomes. Thus, activation of autophagy is often observed in protein aggregation diseases and autophagy deficiency leads to neurodegeneration in mice and fruit flies. The life span of organisms as diverse as *Caenorhabditis elegans *and *Drosophila *and even mice can be significantly increased by boosting autophagy. Efficient autophagy may thus protect against neurodegeneration and increase longevity [1,2].

**Figure 1:**
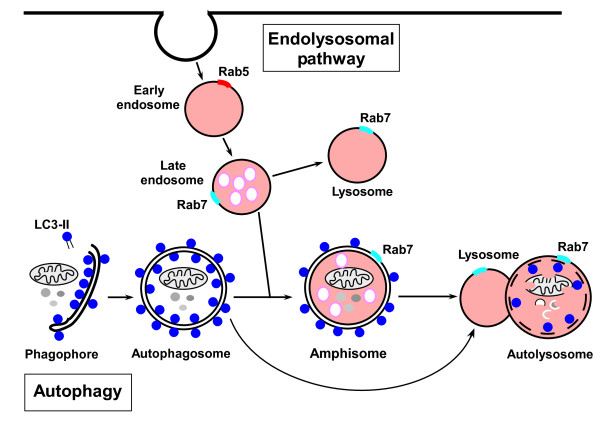
**The autophagic and endolysosomal pathways intersect and interconnect**. Schematic drawing showing the autophagy process with phagophore and autophagosome formation followed by fusion of autophagosomes either directly to lysosomes to form autophagosomes, or to late endosomes to give amphisomes that subsequently fuse with lysosomes. Lipidated LC3-II, used as a marker for autophagy, is tightly associated with the autophagosomes. Note that Rab7, used as a marker of the endolysosomal pathway, is involved in both the autophagic and endolysosomal pathways.

Because of the involvement of autophagy in many disease conditions there is great interest in identifying drugs that can be used to manipulate autophagy for therapeutic purposes. An important step towards this goal is to establish assays and screening systems where the autophagic flux can be measured rapidly and precisely in response to drugs. Autophagic flux refers to the complete process of autophagy (Figure 1), which begins with the formation of a crescent-shaped double membrane, the phagophore, which expands around a portion of cytoplasm and fuses to form the autophagosome. The mature autophagosome then either fuses directly with a lysosome to generate an autolysosome, or fuses first with a late endosome to form an amphisome, which then fuses with the lysosome to give an autolysosome. In the autolysosome, the cargo is degraded.

## Monitoring autophagic flux

It is important to distinguish effects on lysosomal activity from effects on autophagosome formation. This problem has not previously been properly addressed in screening assays used to identify compounds modulating autophagy. The assay described by Hundeshagen and colleagues [5], by the ingenious use of tandem fluorescent tags with differential pH sensitivity, allows this discrimination. The authors use this assay successfully in a secondary screen to identify cardiac glycosides as modulators of autophagy.

Most assays for autophagy modulators use the autophagy marker protein LC3 as readout for autophagic activity. LC3 is a mammalian homolog of the yeast ATG8 protein, a ubiquitin-like protein that becomes lipidated and tightly associated with the autophagosomal membranes. LC3 is also important in the process of selective autophagy, whereby intracellular components such as protein aggregates, organelles and pathogens are removed [3]. Selective autophagy is mediated by autophagic cargo receptors such as p62, NBR1, NDP52 and NIX, which contain an LC3-interacting region (LIR) and can therefore bind directly to LC3 [3]. LC3 proteins are specifically cleaved at their carboxyl termini to form LC3-I, which has an exposed carboxy-terminal glycine that is conjugated to phosphatidylethanolamine to form LC3-II, which is tightly bound to the autophagosomal membranes and serves as an autophagic marker protein.

The most popular of the assays using LC3 are microscopy-based green fluorescent protein (GFP)-LC3 puncta formation assays and western blots of LC3-I and LC3-II forms [6]. Shvets *et al*. more recently pioneered the use of flow cytometry to quantify the turnover of GFP-LC3 as an assay to measure autophagic activity in living mammalian cells [7].

**Figure 2:**
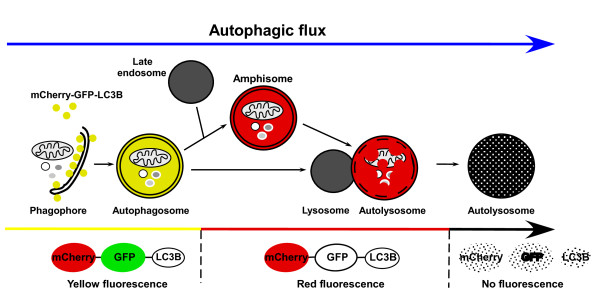
**Tracking different stages of autophagy with double-tagged LC3B**. A tandem fusion of mCherry and GFP is fused to LC3B (one of the several members of the mammalian LC3 family) to make a pH-sensitive sensor that is used to monitor autophagy in live cells. The GFP tag is acid-sensitive while the mCherry tag is acid-insensitive. The double tagged LC3 can be used to label autophagosomes, amphisomes and autolysosomes. In autophagosomes both tags emit fluorescent light resulting in a yellow fluorescence. However, fusion of autophagosomes to late endosomes or lysosomes results in acidic amphisomes or autolysosomes where the green fluorescence from GFP is lost. Subsequently, the red fluorescence from mCherry is lost when the double tagged protein is degraded.

Hundeshagen and colleagues [5] used GFP-LC3B in an initial screen by flow cytometry to quantify autophagosome formation in response to a library of 1,120 Food and Drug Administration approved compounds. The assay uses the human breast cancer cell line MCF-7 stably expressing GFP-LC3B to quantify autolysosome formation measuring GFP fluorescence intensity in 96-well plates, and identified 38 compounds as potential activators and 36 as inhibitors of autophagy. Among the activators were several cardiac glycosides, including digoxin, strophanthidin and digoxigenin, and in the next step, these compounds were screened using a tandem fusion of the red, acid-insensitive mCherry and the acid-sensitive GFP to measure formation of autolysosomes and their degradation by flow cytometry in a large cell population (Figure 2). Because of the low pH of the autolysosome, the green fluorescence from the acid-sensitive GFP is lost on fusion of the autophagosome with the lysosome, but the red fluorescence from the acid-insensitive mCherry is not lost until the proteins are degraded in the autolysosome. The double tag strategy to distinguish autophagosomes from autolysosomes has been described before [8,9], but this is the first report of its use for flow cytometry to monitor distinct steps in the autophagic pathway.

## The significance of cardiac glycosides, and some provisos

A general problem when attempting to measure autophagic flux is the interconnection between the endocytic and autophagosomal pathways (see Figure 1), which makes it difficult to ensure that the observed effects are on autophagic flux and not the endolysosomal pathway. Compounds affecting late steps of the autophagic pathway may also interfere with the endocytic pathway. To address this, Hundeshagen *et al*. [5]used GFP-Rab7 as a marker of endolysosomal activity. Rab7 is associated with late endosomes, lysosomes and autolysosomes and is required for fusion of autophagosomes to lysosomes (see Figure 1) and is therefore not a specific indicator of endolysosomal flux. However, since the authors [5] monitor the fluorescence levels of GFP-Rab7 along with the double tagged LC3, the strategy seems reasonable and can give valuable information on effects on endolysosomal activity.

Using the double tag LC3B and the GFP-Rab7 degradation assays, Hundeshagen *et al*. [5] showed digoxin to be the most potent autophagy stimulator. Cardiac glycosides are used in treatment of heart failure and arrhythmia. These drugs increase the level of calcium by inhibiting the Na^+^/K^+^-ATPase, and thereby increase cardiac contractile force. It is known that increases in intracellular calcium induce autophagy, so it is perhaps not surprising that cardiac glycosides activate autophagy. Cardiac glycosides have been suggested for cancer therapy, and their stimulatory effect on autophagy may be important in this context.

There is one further proviso about screening strategy. Hundeshagen *et al*. [5] used GFP-LC3 for the initial screen and then employed mCherry-GFP-LC3 in a secondary screening, so it is not clear what the outcome would have been if the authors had used mCherry-GFP-LC3 in the primary screen. The importance of the strategy chosen for the primary screen is emphasized by results from another group [10], who screened a collection of 3,584 chemicals, including the chemical library Hundeshagen *et al*. [5] used, and in the same cell line, without identifying the same autophagy modulators. The difference was that Balgi *et al*. [10] used an automated microscopy screen based on the GFP-LC3 puncta formation assay. In this screen perhexiline, niclosamide, amiodarone and rottlerin were identified as autophagy modulators [10]. Considering these different results, and the caveats and shortcomings that different assays have, it seems necessary to use a combination of different assays to perform exhaustive screens for small molecule autophagic modulators.

That said, the work of Hundeshagen and colleagues [5] is clearly a step forward in quantitative cell population based monitoring of distinct steps of the autophagy pathway in the screening for autophagy modulators. Further development and refinement of autophagy screening protocols from this and other groups are to be expected.
